# Brain Injury in Very Preterm Children and Neurosensory and Cognitive Disabilities during Childhood: The EPIPAGE Cohort Study

**DOI:** 10.1371/journal.pone.0062683

**Published:** 2013-05-02

**Authors:** Stéphane Marret, Laetitia Marchand-Martin, Jean-Charles Picaud, Jean-Michel Hascoët, Catherine Arnaud, Jean-Christophe Rozé, Patrick Truffert, Béatrice Larroque, Monique Kaminski, Pierre-Yves Ancel, for the EPIPAGE Study Group

**Affiliations:** 1 Department of Neonatal Medicine, Rouen University Hospital and Region-INSERM Team, ERI 28, NeoVasc, Institute for Biomedical Research and Innovation, Rouen University, Rouen, France; 2 INSERM, UMR S953, Epidemiological Research Unit on Perinatal Health and Women's and Children’s Health, Hôpital Tenon, Paris, France; UPMC Univ Paris 06, UMR S 953, Paris, France; 3 Department of Neonatalogy, Hôpital de la Croix Rousse, Hospices Civils, Lyon, France; 4 Neonatal Department, Maternité Régionale, Nancy, France; 5 INSERM UMR U558, Research Unit on Perinatal Epidemiology, Child Health and Development, University Toulouse III Paul Sabatier, Toulouse, France; 6 Department of Neonatal Medicine, Hôpital Jeanne-de-Flandres, Lille, France; 7 Department of Neonatal Medicine, Nantes University Hospital, Nantes, France; 8 INSERM, UMR S953, Epidemiological Research Unit on Perinatal Health and Women's and Children’s Health, Hôpital Tenon, Paris, France; UPMC Univ Paris 06, Paris, France; Anhui Medical University, China

## Abstract

**Objective:**

To investigate the association of motor and cognitive/learning deficiencies and overall disabilities in very preterm (VPT) children and their relations to gestational age (GA) and brain lesions.

**Design, Setting, and Participants:**

EPIPAGE is a longitudinal population-based cohort study of children born before 33 weeks’ gestation (WG) in 9 French regions in 1997–1998. Cumulating data from all follow up stages, neurodevelopmental outcomes were available for 90% of the 2480 VPT survivors at 8 years. Main outcomes were association of motor and cognitive deficiencies and existence of at least one deficiency (motor, cognitive, behavioral/psychiatric, epileptic, visual, and/or hearing deficiencies) in three GA groups (24–26, 27–28, and 29–32WG) and four groups of brain lesions (none, minor, moderate, or severe).

**Results:**

VPT had high rates of motor (14%) and cognitive (31%) deficiencies. Only 6% had an isolated motor deficiency, 23% an isolated cognitive one and 8% both types. This rate reached 20% among extremely preterm. Psychiatric disorders and epilepsy were observed in 6% and 2% of children, respectively. The risks of at least one severe or moderate deficiency were 11 and 29%. These risks increased as GA decreased; only 36% of children born extremely preterm had no reported deficiency. Among children with major white matter injury (WMI), deficiency rates reached 71% at 24–26WG, 88% at 27–28WG, and 80% at 29–32WG; more than 40% had associated motor and cognitive deficiencies. By contrast, isolated cognitive deficiency was the most frequent problem among children without major lesions.

**Conclusions:**

In VPT, the lower the GA, the higher the neurodisability rate. Cerebral palsy is common. Impaired cognitive development is more frequent. Its occurrence in case without WMI or early motor disorders makes long-term follow up necessary. The strong association between motor impairments, when they exist, and later cognitive dysfunction supports the hypothesis of a common origin of these difficulties.

## Introduction

Very preterm (VPT) infants born before 33 weeks of gestation (WG) are at particularly high risk for a range of neurodevelopmental impairments including cerebral palsy (CP) and sensory, cognitive, and behavioral disabilities. CP is a neurodevelopmental motor condition that can be recognized from early childhood; its prevalence has decreased or at least levelled off over the last decade [1], [2], [3], [4]. The prevalence of cognitive and behavioral deficits, however, remains high [5], [6]. Although these children can suffer from multiple difficulties, not enough is known about the associations between outcomes in different developmental domains.

Although anoxia-ischemia at birth has long been considered the predominant factor, the pathophysiology of neonatal brain injury and subsequent neurodevelopmental disorders is complex and multifactorial. White matter injury (WMI) is classically considered the most reliable prognostic indicator of CP, thought to explain the severest forms of developmental motor disorders [7], [8], [9]. Recent studies, however, show that neuronal/axonal disease is associated with periventricular WMI and affects the subcortical white matter, cortex, basal ganglia, brainstem, and cerebellum [9]. The interrelation between destructive and developmental disturbances is emphasized in the occurrence of sequelae and subsequent handicaps.

Our objective was to improve our understanding of the neurodevelopmental disorders of VPT children and their interplay in various domains. Therefore, we investigated motor and cognitive deficiencies, psychiatric/behavioral disorders, epilepsy, neurosensory deficits, their co-occurrence, and their associations with gestational age at birth and neonatal cranial ultrasonographic (cUS) abnormalities in a large population-based cohort of VPT infants followed through 8 years of age.

## Methods

### Population Study

The Epipage study covered all births between 22 and 32 completed WG in 1997 and all births between 22 and 26 completed WG in 1998 in 9 regions of France and, after parental consent, included all children discharged alive from the hospital. At recruitment in the maternity or neonatal unit, investigators told parents about the study, gave them written information, and obtained verbal consent. Because only 3 infants born at 23 WG survived, the study was limited to children born between 24 and 32 WG. This study is limited to survivors at 8 years of age.

In accordance with French regulations, the study and the verbal consent were approved by the Comité consultatif sur le traitement de l'information en matière de recherche dans le domaine de la santé and the Commission Nationale de l’Informatique et des Libertés (the French data protection agency). There was no ethic approval from ethics comittee because it was not necessary in France for an observational study. The verbal consent was documented in each patient medical file.

### Data Collection

At recruitment, medical and social information was collected in maternity and neonatal units. The follow-up included the following stages:

1. At 2 years, a standard questionnaire was completed by the child's physician [10];
2. At 5 years, a standardized medical examination, including a short version of the Touwen neurologic examination and a developmental evaluation with the Kaufman Assessment Battery for Children (K- ABC), was conducted by trained examiners in special centers set up for the study [6], [11];
3. At 8 years of age, parents received a mail questionnaire asking about the child's health and school situation [12]. In 5 regions, the local office for people with disabilities (Maison Départementale des Personnes Handicapées, MDPH), responsible for meeting specific needs of disabled people at all ages, completed an additional questionnaire, after searching both for children already known to have a neuromotor or sensory deficiency or learning disability or to be in a specialized center or school and for those whose parents did not return the questionnaire at 8 years. Information on deficiencies, special schooling, and special support and aids was collected for all children with a record at their local MDPH.

### Neonatal Characteristics

Gestational age refers to the number of completed weeks of amenorrhea and was studied in the following classes: 24–26, 27–28, 29–30, and 31–32 WG.

During the neonatal period, 97% of the EPIPAGE study infants had at least one cUS in the neonatal period and 66% had at least 3 [13]. Two major types of cerebral lesions were assessed: intraventricular hemorrhage (IVH) with white matter disease (intraparenchymal hemorrhage, IPH), and periventricular leukomalacia (PVL) with ventricular dilatation). Subependymal hemorrhage was classified as grade I, IVH without ventricular dilation as grade II, and IVH with ventricular dilatation as grade III. IPH included a large unilateral parenchymal hyperdense area or large unilateral porencephalic cyst. PVL was defined by the presence of periventricular white-matter echolucent areas (cystic PVL) or echodense spots persisting more than 14 days without cyst formation. Ventricular dilatation was defined by an isolated dilatation of ventricles with no associated IVH. When several cerebral lesions were observed, the most severe was considered. Brain injury was classified as follows: 1) cystic PVL or IPH (major lesions), 2) persistent echodense areas or ventricular dilatation or grade III IVH (moderate lesions), 3) grade II or grade I IVH (minor lesions), 4) no identified cerebral abnormality (no lesion).

### Outcome Indicators (Table 1)

Six deficiencies or disorders were considered: motor deficiencies, cognitive deficiencies/learning disabilities, psychiatric disorders, epilepsy, visual deficiency, and hearing deficiency. To reduce bias due to loss to follow-up and to be able to classify as many children as possible, we used all available data from all stages of follow-up (2, 5, and 8 years) for each deficiency to determine if it affected the child and how severely. Severity was assessed according to the most recent information available. Moreover, when information in one domain of development was missing at one stage of follow up, the child was considered free from this deficiency at that stage.

Two combined indicators were considered. The first describes any combination of motor and cognitive deficiencies, regardless of other deficiencies or disorders. The second assesses the presence of at least one severe deficiency versus at least one moderate deficiency versus none, again regardless of the deficiency or disorder. Table 1 summarizes all deficiency indicators and their definitions.

**Table 1:** Classification of deficiencies.(1)

**Neuromotor deficiencies**	
Severe CP	CP unable to walk or walking only with aid at 8 or 5 years, or 2 years if no further follow-up
Moderate CP	CP walking without aid at 8 or 5 years or 2 years if no further follow-up
No CP, other motor disorder	No CP but MND2 at Touwen examination(2) at age 5
	or dyspraxia or motor coordination trouble (ICD F82, R26, R27) at 8 or 5 years, or 2 years if no further follow-up
None identified	No CP and no motor disorder identified(3)
**Cognitive deficiencies/learning disabilities**	
Severe	Mental retardation at 8 or 5 years (ICD F70–F79)
	or special school/class(4) at 8 years with MPC at 5 years <70(5)
	or no information at 8 years but MPC at 5 years <70
	or mental retardation at 2 years, if no further follow-up
Moderate	Moderate/mild cognitive deficiency mentioned in MDPH(6) questionnaire with no other details,
	or if in a mainstream class at 8 but repeated one grade and/or receiving/needing special support at school(4)
	or no information at 8 years, MPC between 70 and 84 at 5 years
None identified	Mainstream class appropriate for age without any special support at 8 years
	or if no information at 8 years, MPC ≥85 at 5 years
	or if only medical examination at 2 or 5 years, no cognitive deficiency mentioned
**Association of motor and cognitive deficiencies** (7)	
Motor only	Severe or moderate CP or other motor disorder without cognitive deficiency/learning disability
Cognitive only	Severe or moderate cognitive deficiency/learning disability without motor deficiency
Motor and cognitive	Motor deficiency and cognitive deficiency/learning disability associated
None	No motor or cognitive deficiency identified
**Psychiatric disorder**	
Severe	Autism, pervasive development disorders (ICD F84) at 8 or 5 years
Moderate	Hyperactivity or attention deficit disorder (ICD F90)
	or conduct disorder (ICD F91) as reason for a visit to a psychiatrist or a psychologist at 5 or 8 years
None identified	None of the above
**Epilepsy**	Reported at 8, 5 or 2 years (ICD G40)
	or antiepileptic drug treatment reported at 8 or 5 years
**Visual deficiency**	Blindness (uni- or bilateral)
	or Rossano test ≤2 in both eyes at 5 years
**Hearing deficiency**	Deafness in one or both ears
	or use of hearing aid at any age
**Overall deficiencies**	
Severe	At least one of: severe CP, severe cognitive deficiency/learning disabilities,
	severe psychiatric disorder, epilepsy, visual deficiency or hearing deficiency
Moderate	At least one of: moderate CP, other motor disorder, moderate cognitive deficiency
	or moderate psychiatric disorder
None identified	None of the above

(1) For each deficiency, the classification follows a priority order according to severity at the most recent step of the follow-up available.

(2) Moderate Neuromotor Dysfunction (MND-2) at the short version of the Touwen neurological examination at the age of 5 years ^11.^

(3) Including children free of CP or other neuromotor disorders but who were not assessed with the Touwen examination.

(4) Except for visual or hearing deficiency only; for 25 children in special school/class at 8 years of age with MPC at 5 years ≥70 or missing, other data in their record allowed us to classify 14 with a severe cognitive deficiency, 10 with moderate, and 1 with none identified record allowed to classify 14 as severe crecords.

(5) MPC = Mental Processing Composite of the K-ABC test ^14.^

(6) MDPH = Maisons départementales des Personnes handicapées.

(7) Regardless of all other deficiencies.

### Statistical Analysis

Results are presented as proportions of the number of survivors included in the follow-up after discharge from neonatal care. Deficiencies and their grouping were reported according to gestational age and categories of neonatal brain injuries diagnosed by ultrasonographic studies. We used the chi^2^ test to compare outcomes between the different gestational age groups of VPT children. Finally the neonatal characteristics of children completely lost to follow-up were compared with those for whom we had at least one stage of follow-up. Statistical analyses were performed with SAS software (version 9.2).

## Results

Of the 2480 children born at 24–32 WG who survived to the age of 8 years, we obtained information at 2 years of age for 2055 (83%) (Table 2). At 5 years, 1897 (76%) children had a medical examination, and 1594 (64%) were assessed with the K-ABC [14]. At age 8, we had a parental questionnaire or one completed by the MDPH or both for 1617 (65%) of the VPT children. Data obtained at any step of the follow-up provided information for 2220 (90%) of the eligible VPT children, but 170 children were followed only to the age of 2 years (Table 2). Overall, 10% of the children (n = 260) were lost to follow-up. They did not differ significantly for cUS lesions from those with follow-up (19% in each group had a major or moderate neonatal cerebral lesion). Although they had a slightly higher gestational age at birth than those included in the study (9% of births <27 weeks compared to 12%), this difference was not significant. By contrast, their families belonged to less privileged social classes, 64% working in service and manual occupations, compared to 38% of those who continued to participate.

**Table 2:** Population study.

	24–32 weeks		24–26 weeks	
	Inclusion 1997		Inclusion 1998	
Discharged alive	2382		128	
Deaths between discharge and 8 years	27		3	
**Survivors at 8 years**	**2355**		**125**	
Medical examination at 2 years	1949	83%	106	85%
Medical examination at 5 years	1811	77%	86	69%
* with K-ABC test* (2)	*1533*	*65%*	*61*	*49%*
Parental questionnaire and/or MDPH at 8 years	1531	65%	86	69%
**Follow-up at least at one age**	**2109**	**90%**	**111**	**89%**
* only follow up at 2 years*	*159*		*11*	
Lost to follow up	246	10%	14	11%
* Because of refusal to participate refusals*	*106*		*6*	

(1) All percentages calculated among survivors at 8 years.

(2) Kaufman Assessment Battery for Children ^14.^

The VPT children had high rates of motor (14%) and cognitive (31%) deficiencies (Table 3). Rates of both deficiencies increased as gestational age decreased and reached 32% and 48% respectively at 24–26 WG. Only 6% of children had a motor deficiency with no cognitive problems, whereas 23% had a cognitive deficiency without a motor deficiency, and 8% had both. The rate of both combined reached 20% among extremely preterm children (24–26 WG). Moderate and severe psychiatric disorders, ie, autism, attention-deficit/hyperactivity, and conduct disorders, were observed in 6% and 0.5% of VPT children respectively. Two percent had epilepsy.

**Table 3:** Deficiencies according to gestational age.

	24–32 weeks(1)		24–26 weeks(2)		27–28 weeks		29–30 weeks		31–32 weeks		p-value
	n = 2109	%	n = 261	%	n = 349	%	n = 556	%	n = 1054	%	
**Neuromotor deficiencies**											
Severe CP	66	3.1	19	7.3	16	4.6	19	3.4	21	2.0	<0.001
Moderate CP	124	5.9	34	13.0	31	8.9	28	5.0	45	4.3	
No CP, other motor disorder	110	5.2	31	11.9	23	6.6	31	5.6	35	3.3	
None identified	1809	85.8	177	67.8	279	79.9	478	86.0	953	90.4	
**Cognitive deficiencies/learning disabilities**											
Severe	137	6.5	23	8.8	36	10.3	36	6.5	52	4.9	<0.001
Moderate	518	24.6	102	39.1	103	29.5	120	21.6	237	22.5	
None identified	1454	68.9	136	52.1	210	60.2	400	71.9	765	72.6	
**Association of motor and cognitive deficiencies**											
Motor only	138	6.5	31	11.9	22	6.3	47	8.5	51	4.8	<0.001
Cognitive only	493	23.4	72	27.6	91	26.1	125	22.5	239	22.7	
Motor and cognitive	162	7.7	53	20.3	48	13.8	31	5.6	50	4.7	
None	1316	62.4	105	40.2	188	53.9	353	63.5	714	67.7	
**Psychiatric disorder**											
Severe	11	0.5	1	0.4	7	2.0	1	0.2	3	0.3	<0.001
Moderate	120	5.7	23	8.8	27	7.7	29	5.2	48	4.6	
None identified	1978	93.8	237	90.8	315	90.3	526	94.6	1003	95.2	
**Epilepsy**											
Yes	45	2.1	8	3.1	10	2.9	10	1.8	20	1.9	0.47
No	2064	97.9	253	96.9	339	97.1	546	98.2	1034	98.1	
**Visual deficiency**											
Yes	19	0.9	7	2.7	3	0.9	2	0.4	10	0.9	0.019
No	2090	99.1	254	97.3	346	99.1	554	99.6	1044	99.1	
**Hearing deficiency**											
Yes	17	0.8	7	2.7	4	1.1	2	0.4	8	0.8	0.012
No	2092	99.2	254	97.3	345	98.9	554	99.6	1046	99.2	
**Overall deficiencies**											
Severe	230	10.9	50	19.2	59	16.9	60	10.8	86	8.2	<0.001
Moderate	616	29.2	117	44.8	112	32.1	155	27.9	277	26.3	
None identified	1263	59.9	94	36.0	178	51.0	341	61.3	691	65.6	

(1) Inclusion 1997.

(2) inclusion 1997+1998.

The risk of having at least one severe or moderate deficiency of any kind was 11 and 29%, respectively, and it decreased as gestational age increased: Only 36% of children born extremely preterm had no reported deficiency compared to 51% at 27–28 WG, 61% at 29–30 WG, and 66% at 31–32 WG. Rates of CP (6%) and other motor disorders (4%) were lower for the 170 children followed only to 2 years of age than among the 2050 children with a longer follow-up (10% and 6% respectively). Little information was available about the cognitive deficiencies and psychiatric status of those 170, understandably given their age (results not shown).

Rates of overall and severe deficiencies were high among children with major WMI: 71% and 42% respectively at 24–26 WG, 88% and 50% at 27–28 WG, and 80% and 53% at 29–32 WG (Table 4). More than 40% had associated motor and cognitive deficiencies. However, 19 children with major WMI had no identified sequelae: 2 had a follow up at 2 years of age only, 7 at 5 years, and 10 through 8 years. In all 19, the major brain injury was located in only one area: frontal, occipital, parietal or other.

**Table 4:** Deficiencies according to gestational age and neonatal cerebral lesions.

LESIONS																								
	24–26 weeks(1)								27–28 weeks(2)								29–32 weeks(2)							
	Neonatal cerebral lesions(3)								Neonatal cerebral lesions(3)								Neonatal cerebral lesions(3)							
	Major		Moderate		Minor		None		Major		Moderate		Minor		None		Major		Moderate		Minor		None	
	n = 24	%	n = 72	%	n = 68	%	n = 96	%	n = 26	%	n = 85	%	n = 70	%	n = 168	%	n = 47	%	n = 183		n = 220	%	n = 1128	%
**Neuromotor deficiencies**																								
Severe CP	7	29.2	5	6.9	2	2.9	5	5.2	7	26.9	4	4.7	3	4.3	2	1.2	20	42.6	7	3.8	2	0.9	10	0.9
Moderate CP	5	20.8	9	12.5	11	16.2	9	9.4	8	30.8	10	11.8	4	5.7	9	5.4	9	19.1	17	9.3	13	5.9	34	3.0
No CP, other motor disorder	2	8.3	12	16.7	8	11.8	9	9.4	2	7.7	7	8.2	2	2.9	12	7.1	4	8.5	11	6.0	15	6.8	36	3.2
None identified	10	41.7	46	63.9	47	69.1	73	76	9	34.6	64	75.3	61	87.1	145	86.3	14	29.8	148	80.9	190	86.4	1048	92.9
**Cognitive deficiencies/learning disabilities**																								
Severe	6	25.0	7	9.7	5	7.4	5	5.2	8	30.8	15	17.6	7	10.0	6	3.6	13	27.7	13	7.1	15	6.8	46	4.1
Moderate	6	25.0	26	36.1	33	48.5	36	37.5	10	38.5	29	34.1	19	27.1	45	26.8	12	25.5	39	21.3	37	16.8	262	23.2
None identified	12	50.0	39	54.2	30	44.1	55	57.3	8	30.8	41	48.2	44	62.9	117	69.6	22	46.8	131	71.6	168	76.4	820	72.7
**Association of motor and cognitive deficiencies**																								
Motor only	4	16.7	13	18.1	5	7.4	9	9.4	4	15.4	6	7.1	3	4.3	9	5.4	13	27.7	21	11.5	20	9.1	43	3.8
Cognitive only	2	8.3	20	27.8	22	32.4	27	28.1	5	19.2	29	34.1	20	28.6	37	22.0	5	10.6	38	20.8	42	19.1	271	24.0
Motor and cognitive	10	41.7	13	18.1	16	23.5	14	14.6	13	50.0	15	17.6	6	8.6	14	8.3	20	42.6	14	7.7	10	4.5	37	3.3
None	8	33.3	26	36.1	25	36.8	46	47.9	4	15.4	35	41.2	41	58.6	108	64.3	9	19.1	110	60.1	148	67.3	777	68.9
**Psychiatric disorder**																								
Severe	0	0	0	0	0	0	1	1.0	1	3.8	3	3.5	2	2.9	1	0.6	1	2.1	0	0	1	0.5	2	0.2
Moderate	2	8.3	3	4.2	9	13.2	9	9.4	2	7.7	9	10.6	8	11.4	8	4.8	3	6.4	6	3.3	13	5.9	54	4.8
None identified	22	91.7	69	95.8	59	86.8	86	89.6	23	88.5	73	85.9	60	85.7	159	94.6	43	91.5	177	96.7	206	93.6	1072	95
**Epilepsy**																								
Yes	1	4.2	2	2.8	1	1.5	4	4.2	4	15.4	1	1.2	3	4.3	2	1.2	9	19.1	6	3.3	5	2.3	9	0.8
No	23	95.8	70	97.2	67	98.5	92	95.8	22	84.6	84	98.8	67	95.7	166	98.8	38	80.9	177	96.7	215	97.7	1119	99.2
**Visual deficiency**																								
Yes	1	4.2	1	1.4	3	4.4	2	2.1	0	0	1	1.2	1	1.4	1	0.6	4	8.5	1	0.5	2	0.9	5	0.4
No	23	95.8	71	98.6	65	95.6	94	97.9	26	100	84	98.8	69	98.6	167	99.4	43	91.5	182	99.5	218	99.1	1123	99.6
**Hearing deficiency**																								
Yes	0	0	0	0	3	4.4	4	4.2	0	0	0	0	3	4.3	1	0.6	0	0	2	1.1	1	0.5	7	0.6
No	24	100	72	100	65	95.6	92	95.8	26	100	85	100	67	95.7	167	99.4	47	100	181	98.9	219	99.5	1121	99.4
**Overall deficiencies**																								
Severe	10	41.7	12	16.7	12	17.6	16	16.7	13	50.0	20	23.5	14	20.0	12	7.1	25	53.2	22	12.0	24	10.9	73	6.5
Moderate	7	29.2	35	48.6	36	52.9	38	39.6	10	38.5	31	36.5	20	28.6	51	30.4	13	27.7	54	29.5	54	24.5	304	27.0
None identified	7	29.2	25	34.7	20	29.4	42	43.8	3	11.5	34	40.0	36	51.4	105	62.5	9	19.1	107	58.5	142	64.5	751	66.6

(1) inclusion 1997+1998.

(2) inclusion 1997.

(3) Major : cystic PVL or IPH, Moderate : persistent echodensities or ventricular dilatation or grade III IVH, Minor : grade II and grade I IVH (1 missing neonatal cerebral lesion information in the group of 24–26 SA and 32 missing in the groups 29–32 SA).

Inversely, isolated cognitive deficiency was much more frequent than either isolated motor deficiencies or the combination of motor and cognitive deficiencies among children with moderate or minor cerebral injury, as well as those with none (Table 4). The global risk of any deficiency remained high among extremely preterm children without major WMI (56%), but decreased as gestational age increased (Table 4). Finally, 101 VPT children without identified cerebral lesions developed severe deficiencies; all but one had had at least one cUS and 85% 2 or more. Most (57) had a severe cognitive deficiency, while 17 had severe CP.

## Discussion

This study shows a high rate of neurodisabilities in VPT children: even among those with no WMI on neonatal cUS, around 40% had at least one severe or moderate deficiency. The lower the gestational age, the higher the neurodisability rate. Cognitive deficiencies without motor disorders were more frequent than either combined motor and cognitive deficits or isolated motor deficiencies. Nonetheless, these combined deficiencies were frequent among extremely preterm children and those with major cUS WMI. Cognitive deficiencies without motor deficit were predominant among children with minor/moderate or no cUS brain injury and provide evidence of impaired brain development in these children.

The EPIPAGE study is the largest population-based study investigating the outcome of very preterm children born before 33 WG since the Bavarian study and the POPS study in the Netherlands in the mid-1980s [15], [16]. Information collected from 2 to 8 years of age supplied neurodevelopmental status for 90% of the eligible VPT children; it was available for 80% at 2 years of age, 77% at 5 years, and 64% at 8 [6], [10], [12]. This follow up rate is close to those for other population-based studies (POPS, EPICure1) [16], [17], [18], especially in view of the large number of children included and the families' substantial geographical dispersion and mobility.

Children lost to follow-up had a slightly higher gestational age at birth than those included in our study, but no difference between the groups was observed for neonatal cerebral lesions. Accordingly, the impact of attrition on the CP rate should be low. However, 170 (7.7%) children were assessed only at 2 years of age, and assessment at an older age would probably have provided a more accurate measure of motor status: motor deficiencies were less frequent in this group than among children followed to the ages of 5 and 8 years. The small number of children assessed only at age 2 probably means that the risk of CP/motor deficits is underestimated only marginally.

Although cognitive deficiencies and psychiatric disorders were frequent among VPT children, methodological issues may nonetheless have induced underestimation: i) we could not assess the cognitive performance of the 170 children followed up only at 2 years; ii) loss to follow-up was more common in socially disadvantaged children, a factor known to be associated with lower cognitive scores; iii) our definition did not consider some kinds of special care widely prescribed by the children's physicians, such as speech or psychomotor therapy. Therefore we have likely missed some learning difficulties not assessed with the Kaufmann Assessment Battery for Children [14], such as dyslexia, dyscalculia, or executive dysfunctions; iv) an 8-year follow-up is not enough to observe specific cognitive problems in VPT children. In 11-year-old extremely preterm children born in England in 1995, Johnson et al^18^ observed that almost two thirds required additional support at school. The impact of impairments increases over time, as cognitive demands grow in parallel with progressively more complex academic studies in secondary school and exacerbate the children's difficulties.^2.^

Eleven percent of all VPT children had at least one severe neurodisability and 29% a moderate one. Rates were inversely correlated with gestational age at birth: 60% of all VPT children had no reported deficiency, but only 36% of those born at 24–26 weeks. This last rate is not very different from the EPICure2 study. Even if definition of disability is not identical, 34% of infants born at 24 and 25 weeks in 2006 were free of disability at three years of age. These results highlight the severity of the prognosis at extremely low gestational ages and raise questions about appropriate care at birth in this population [17], [18], [19]. Another important result concerns cognitive neurodisabilities (31%), which were much more frequent than CP and other motor deficiencies (14%). This high rate justifies medical/clinical follow up of VPT children after 2 years of age.

We previously showed that major cerebral lesions (ie, WMI) were the most important predictor of CP and severe cognitive impairments in these VPT children [20], [21]. Nonetheless, cerebral lesions do not systematically result in developmental problems. In our study, 19 of 97 VPT children with major destructive cUS lesions had no deficiency. All 19 had a major cerebral lesion, but it was located in only one area. Ten of them (50%) were followed through the age of 8 years and had no reported deficiency. Although these results should be interpreted with caution, they suggest that a small proportion of children surviving with a major but localized cerebral lesion might have no deficiency.

The absolute number of CP diagnoses in the group without cUS brain injury was as high as in the group of VPT children with major neonatal cUS brain injury [10] Nonetheless, the children with normal cUS who developed cerebral palsy were less disabled than those with cUS abnormalities and we cannot rule out the possibility that some neonatal cerebral lesions were missed: cUS was conducted according to each hospital's routine practice without any standardized protocol. We also observed that the frequency of moderate cognitive deficit did not differ between children with moderate or minor cUS lesions and those with none. These data suggest that focal destructive brain lesions are not the sole cause of neurodisabilities. The predominance of cognitive versus motor deficiencies, their frequent association, their increase with decreasing gestational age, and the lack of association between moderate cognitive deficiencies and cUS brain injuries all suggest that preterm birth impairs the genetically determined program of corticogenesis in the developing brain.

Cognitive and motor disorders, especially severe ones, can be observed when cUS does not identify destructive parenchymal white matter lesions. Although too few of these examinations might explain this result, it is unlikely, as 96% of children had at least one cUS and 66% three or more [13]. Rather, this finding appears to be evidence of impaired development of the dendritic connections and cortical/subcortical circuits involved in the cerebral cortex as well as the thalami and basal ganglia. Longitudinal analyses with functional connectivity magnetic resonance imaging (fcMRI) have shown prominent differences between networks identified in term control versus premature infants at term equivalent, including in the thalamo-cortical network [22], [23]. In another fcMRI study, Petersen et al [24] detected aberrant semantic processing during a language comprehension task in preterm children. Gozzo et al [25] confirmed that preterm children use different circuits for auditory language processing at school age than term controls.

Independent factors besides preterm birth that are associated with impaired brain development include chronic lung disease, infection, suboptimal growth due to intrauterine growth failure, severe neonatal morbidity, neonatal undernutrition, pre- and postnatal corticosteroid therapy, and pain and stress during neonatal hospitalization [2], [26]. Moreover, cognitive achievement throughout life is influenced by social environment and socioeconomic status [12], [27].

This study also confirms that severe (autism spectrum disorders) and moderate psychiatric disorders (attention deficit/hyperactivity and behavioral disorders) as well as epilepsy are more frequent than in the general population and are inversely correlated with gestational age at birth [28], [29]. Late-migrating GABAergic neurons are a transient neuronal population in the subcortical white matter of the preterm infant, and the decrease in GABAergic neurons (which mediate inhibition of action potential in adults) documented in the central white matter of preterm children with PVL suggests an inhibitory deficit in infancy [9]. However, the lack of an obvious association between psychiatric disorders and neonatal brain injuries once again implies impaired brain development and connectivity in the preterm population. In autism, diffusion tensor imaging tractography has shown differences in the anatomy of frontostriatal white matter tracts in autism, and fcMRI [30], [31] has shown underconnectivity.

In conclusion, we showed here that infants born VPT are at high risk of developing cognitive and motor impairments, a risk inversely correlated with gestational age. The strong association between motor impairments and later cognitive dysfunction supports the hypotheses of a common origin of these difficulties and/or the influence of motor development on subsequent cognitive skills. Improvement in our ability to protect the developing brain is urgently needed as we have no definitive strategy for preventing the long-term neurological consequences that can occur in these infants. Only one compound, low-dose magnesium sulphate given to women at risk of preterm birth, has demonstrated some beneficial effects in the prevention of cerebral palsy in preterm humans [32]. Neonatal developmental care and post-discharge interventions in VPT children must be evaluated over the long term to determine those most effective in improving cognitive and motor development.
